# *Elymus repens* (L.) Gould Phytochemistry Pharmacological Activities and Therapeutic Potential with Future Perspectives

**DOI:** 10.3390/ijms27114928

**Published:** 2026-05-29

**Authors:** Zhakipbekov Kairat Saparkhanovich, Serikbayeva Elmira Asilbekovna, Tleubayeva Meruyert Ilyasovna, Anarbayeva Rabiga Mutalievna, Shimirova Zhanar Kasymbekovna, Umurzakhova Galiya Zhanburbaevna, Seitova Zhanerke Dauletkyzy, Mukanova Arailym Beybitkyzy, Konash Nyshanbay Yermekuly, Ashirov Murat Zulpidinovich, Zulpidin Gulsezim Mukhankyzy

**Affiliations:** 1 School of Pharmacy, Asfendiyarov Kazakh National Medical University, 94, Tole Bi Str., 050012 Almaty, Kazakhstan; zhakipbekov.k@kaznmu.kz (Z.K.S.); elmira.asyl@mail.ru (S.E.A.); meruert_iliasovna@mail.ru (T.M.I.); seitova.zh@kaznmu.kz (S.Z.D.); rai_m93@mail.ru (M.A.B.); gulsezim.zulpidin@mail.ru (Z.G.M.); 2 Faculty of Pharmacy, South Kazakhstan Medical Academy, Al-Farabi Square, 1, 160019 Shymkent, Kazakhstan; rabiga.rm@mail.ru (A.R.M.); shimirova_z@mail.ru (S.Z.K.); galiaum@mail.ru (U.G.Z.)

**Keywords:** *Elymus repens* (L.), phytochemistry, pharmacological effects, diuretic activity, antiurolithiatic, urinary tract disorder

## Abstract

*Elymus repens* (L.) Gould, commonly known as couch grass, is mainly distributed as a medicinal herb of great ethnopharmacological importance. This review presented for the first time the significance of nomenclature clarity, current insights on distribution, traditional use, phytochemistry and their biological activities. The bioactive compound composition of *E. repens* displayed a variety of compounds including carbohydrates, phenolic acids, flavonoids, benzoxazinoids and volatile compounds, all of which contributed to its medicinal properties. The biological potential of this plant underlines diuretic, antiurolithiatic, anti-inflammatory, antioxidant and also antidiabetic qualities with molecular insights showing synergistic interactions across many compound classes. The current reported data notably confirmed its potential in lowering renal calculi and alleviating associated symptoms; however, the findings remain limited. *E. repens*’ medicinal potential is further confirmed by its historical application as an herbal tea and aqueous preparation, which clearly indicates its favorable safety profile. Along with this, from many encouraging results, there exist considerable deficiencies in standardization, clinical validation and also mechanistic clarification. This review highlights the necessity for careful studies to determine the therapeutic efficiency, enhance formulation and promote *E. repens* as a scientifically therapeutic agent.

## 1. Introduction

Medicinal plants have been used by humans for healthcare for many years. The history goes back to 2600 BCE or more, yet many traditionally used plants are still unexplored or incompletely characterized, despite their long history. Even today around 80% of people are using them against many diseases [1,2]. The combination of traditional knowledge with modern scientific approaches has a critical role in discovering and the development of a drug, especially from plants with established safety profiles—which showed long-standing cultural acceptance. Many herbal-derived compounds have been used against many diseases and play a key role in controlling and managing a wide range of pathological conditions [3,4]. There are many herbal remedies available that continue to be marginalized in conventional medical practice, not because of ineffective evidence but because of the fragmented literature, poor standardization and limited rigorous clinical investigation [5]. Natural bioactive compounds played a fundamental role in drug discovery, as they provide structurally distinct compounds that form the basis for therapeutic development. In particular, plant-derived compounds such as flavonoids, alkaloids, terpenoids, and phenolic displayed many biological activities, including antibacterial, antioxidant, antiviral and anticancer, making them effective candidates for the development of modern multi-targeted drugs [4,6,7,8]. Additionally, with the discovery and widespread adoption of synthetic pharmaceuticals for the treatment of numerous diseases, the development and utilization of medicinal plants have not advanced to a comparable level in modern clinical practice [9]. This gap between traditional use and scientific validation resulted in huge opportunities for interdisciplinary research that can either confirm or contest traditional claims while identifying bioactive compounds and mechanism of action. Accordingly, the systematic synthesis of ethnopharmacological knowledge with modern analytical, pharmacological and clinical methodologies is key to achieve the complete therapeutic potential of medicinal plants. These initiatives will not only link ancient practice with modern medicine but will also promote the identification of innovative, safe and effective plant-based pharmaceuticals [10,11].

*Elymus repens* (L.) Gould, commonly known as couch grass or quackgrass, is a valuable example of a traditionally important medicinal plant assessed thorough scientific evaluation [12,13]. While widely recognized as an invasive weed in agriculture contexts, the rhizome possesses significant medicinal importance in traditional European and Asian herbal systems. The long-standing traditional use of *E. repens* rhizome in the management of urinary disorders, along with antioxidant and anti-inflammatory effects, glucose-lowering potential, and lipid-modulating properties provides a strong ethnopharmacological foundation for its modern scientific investigation suggesting further pharmacological and clinical studies to validate its efficacy and elucidate its underlying mechanisms of action [14,15,16,17]. Recent studies in phytochemical profiling, particularly through modern analytical methods like untargeted mass spectrometry, have shown the complex diversity of phytochemicals in *E. repens* rhizome, which indicates that aqueous preparations cover a significant number of bioactive compounds beyond traditional phenolics. The phytochemical analysis of *E. repens* reveals the presence of many allelopathic and phytotoxic compounds, including benzoxazinoids and indole-based compounds, representing their ecological role in plant interactions. Additionally, a broad spectrum of identified compounds, such as flavonoids, phenolic esters, alkylresorcinols, and tryptophan-derived compounds, show mixed biological activities, highlighting the plant’s potential as a significant source of pharmacologically active agents [13,15].

The main aim of this review is to combine the current knowledge regarding the phytochemical composition, pharmacological properties, medicinal significance, and pharmaceutical potential of *E. repens*, specifically focusing on the rhizome, aqueous extracts, and the connection between phytochemicals and biological effects. This review combines ethnomedicinal practices with modern phytochemistry and pharmacology to explain research goals, describe research gaps, and suggest future pathways for the pharmacological advancement of *E. repens*.

## 2. Methodology

A thorough literature search was conducted to retrieve relevant information on *E. repens* and its pharmacological and phytochemical properties. Scientific articles were taken from Web of Science, Google Scholar, PubMed, ScienceDirect, and Scopus. The key words were “*Elymus repens*,” “*Elytrigia repens*,” “*Agropyron repens*,” “medicinal plants,” “phytochemistry,” “biological activity,” “urinary disorders,” “allelopathic compounds,” applied individually or in combination using Boolean operators (“AND,” “OR”).

Peer reviewed articles, review papers and related book chapters published in English were considered. Studies were screened based on their relevance to the scope of the review with special focus on phytochemical composition, traditional use and pharmacological activities. Duplicates, non-relevant studies and articles with no scientific data were excluded. The selected literature was carefully analyzed to provide a comprehensive and critical review of current knowledge on *E. repens*.

## 3. Botanical Identity, Taxonomy, and Nomenclature

### 3.1. Taxonomic Classification and Nomenclature

*Elymus repens* (L.) Gould is the currently accepted name for this species within the Poaceae family according to contemporary taxonomic databases and the World Flora Online (WFO). Several synonymous names, including *Elytrigia repens* (L.) Desv. ex Nevski, *Agropyron repens* (L.) P. Beauv., and *Triticum repens* L., have historically been used in the phytochemical, pharmacological, ethnobotanical, and pharmaceutical literature [12,18,19]. These synonyms are included in this review not as an exhaustive taxonomic synonymy list, but because they represent the most commonly cited names under which important earlier studies were published. The National Agricultural Library Thesaurus Concept Space (NALT) additionally reports related synonymous designations such as *Agropyron firmum* and *Triticum firmum* [19]. This nomenclatural complexity has important implications for literature retrieval and research synthesis, as database searches performed exclusively under the currently accepted name may overlook significant earlier investigations published under historical synonyms.

To eliminate any taxonomic uncertainty, the plant material used in this study was identified and independently confirmed by Dr. Polina Vasilievna Veselova (Head of the Laboratory of Higher Plant Flora, Kazakhstan) and supported by authenticated voucher specimens deposited in an official herbarium repository.

**Figure 1:**
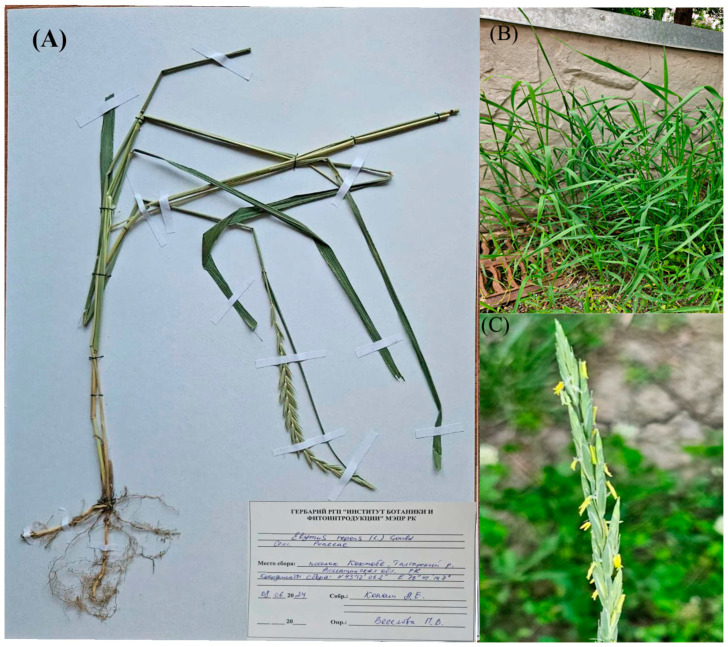
Morphological characteristics of *E. repens*. (**A**) Herbarium specimen showing the rhizomes, root, leaves and spike inflorescence. (The aerial and underground parts of *E. repens* were collected 4 km from Koktyube village, Talgar district, Almaty region (N 43°12′06.2″; E 076°41′14.7″). The collection of plant material was carried out in June 2024. The aerial parts were separated from the rhizomes and dried in accordance with Good Agricultural and Collection Practice (GACP) standards. After drying, the total dry weight was 1000 g of raw materials. Identification of plant material was carried out by Bilibayeva B.K., researcher from the Higher Plant Flora laboratories of the Republican State Enterprise “Institute of Botany and Phytointroduction”. Confirmation of the authenticity of the specimens was issued by official certificate No. 01-05/331 dated 21 June 2024 (index №. 411). The collected plant material was placed for storage in the botanical repository of the National Center for Natural Products Research (NCNPR) of the University of Mississippi, USA, under NCNPR # 26550.) (**B**) Fresh vegetative part in dense clonal habitat. (**C**) Close-up of the spike inflorescence.

Figure 1 illustrates many morphological forms of *E. repens*.

### 3.2. Common Names and Medicinal Part

*E. repens* is known by numerous common names in different languages and regions, including couch grass, quackgrass, wheatgrass, creeping quackgrass, twitch grass, dog grass and various regional vernacular terms. Despite its reputation as an agricultural pest, its rhizome part has medicinal properties and has maintained consistent recognition as a valuable therapeutic agent in traditional medicine systems [19,20]. The rhizome has creeping growth patterns and a high concentration of bioactive compounds. A comprehensive understanding of the medicinal aspects is important, as various plant parts (leaves, stems, roots, rhizomes) may have different qualitative and quantitative phytochemical profiles [13,21,22]. Current pharmaceutical development should focus on rhizome, as it is both the traditional medicinal component and the most thoroughly characterized in terms of chemical and pharmacological properties.

### 3.3. Importance of Nomenclatural Clarity

The taxonomic history of *E. repens* has resulted in a fragmented literature, with older phytochemical and pharmacological studies frequently published under the synonymous names *Agropyron repens* and *Triticum repens* [14,23,24]. There is a need for comprehensive literature systematic studies that include all these botanical synonyms to cover all missing important studies. This nomenclatural complexity represents both a challenge and an opportunity; while it confuses literature synthesis, it also means that research databases may contain substantial unpublished or underutilized data on this plant that could be recovered through careful bibliographic reconstruction. Table 1 shows the botanical identity and traditional uses of *E. repens*.

## 4. Distribution, Traditional Use, and Ethnomedicinal Relevance

### 4.1. Geographic Distribution and Ecological Status

*E. repens* (synonym: *Elytrigia repens*) is a plant native to temperate regions of the Euro-Mediterranean region, including Macaronesia, the Black Sea region, the Caucasus, Siberia, the Russian Far East, Central and Western Asia, as well as the Himalayas, China, Korea and Mongolia. The species has also been widely introduced outside its native range, including Japan, South Africa, North America (including Alaska and Greenland), Mexico, parts of South America (especially Peru and the Southern Cone), Australia and New Zealand. Due to its high ecological adaptability, it is recognized as a valuable forage grass in some areas, while in others it is considered an invasive weed [22,23]. Its invasive properties in agricultural environments are associated with its rapid rhizome propagation and environmental tolerance, which allow it to spread far beyond its native range. Its wide geographical distribution demonstrates its ecological diversity, and it is recognized as a well-documented plant species in different environments. Furthermore, its widespread presence has allowed it to be incorporated into various regional traditional medicine systems, indicating that its ethnomedicinal uses emerged through independent empirical observations in different civilizations, and not simply as a result of cultural transmission [24,25].

### 4.2. Traditional Medicinal Uses

*E. repens* has a long history of traditional use for many purposes. The rhizome of *E. repens* is traditionally used as a mild diuretic, promoting improved urine production without electrolyte loss. It is also used as a urinary sedative to relieve pain and spasm of the urinary tract, which mainly targets symptoms related to urinary tract issues [26,27,28]. The mucilaginous properties of *E. repens* show demulcent properties; also, it is used as a general tonic, which shows its nutritional values [23]. It is also used for urinary gravel and stone problems by many traditional practitioners, which suggests that *E. repens* has anticalculus or antiurolithiatic activities [29,30]. Based on these traditional uses, this plant forms a solid framework that modern research can study using modern biological techniques.

## 5. Phytochemistry of *E. repens*

### 5.1. Overview of Phytochemical Diversity and Complexity

The rhizome of *E. repens* contains both primary and secondary metabolites, with its chemical makeup differing according to the plant part, harvesting season, geographic origin, and extraction method. The rhizome is distinguished by significant phytochemical complexity, containing structural classes that differ from simple monosaccharides to complex polyphenolic compounds. The reported phytochemicals are fructans and other carbohydrates, mucilage and pectin, phenolic acids, flavonoids, volatile compounds, amino acids and indole-related substances, benzoxazinoids and other allelochemicals, as well as inorganic ingredients such as minerals, silica, and iron [15,31]. This variation in chemical makeup shows that *E. repens*’ biological activities have resulted from the combined action of many compound classes instead of single major compounds. Understanding this complexity will be important for pharmaceutical development, as it shows that standardization strategies should be considered, and the activities cannot be attributed to single isolated compounds.

### 5.2. Carbohydrates, Fructans, Mucilage, and Polysaccharides

Rhizome is especially abundant in water-soluble polar compounds, making aqueous formulations pharmacologically significant and clarifying the conventional use of herbal tea. Triticin, a fructosan-type polysaccharide, has been found as a principal component among the carbohydrate elements, along with fructosan, inulin, and simpler sugars such as fructose, glucose, and rhamnose [15]. The rhizome also consists of different disaccharides and trisaccharides, increasing its reported sweetness. In addition to simple and oligomeric sugars, the rhizome contains mucilaginous compounds and pectin, which significantly improve the demulcent and calming properties traditionally related to *E. repens* [15,23].

**Figure 2:**
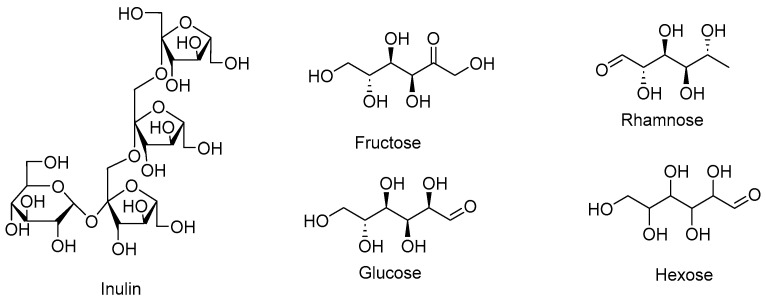
Structures of carbohydrates found in *E. repens*.

Modern analytical techniques have confirmed these classical observations about carbohydrate composition. Recent untargeted ESI-MS/MS profiling of herbal tea showed the presence of hexoses, di-hexoses, and tri-saccharide-type constituents, supporting and extending older phytochemical reports of fructan-rich rhizomes. The abundance of saccharides and fructan-type constituents suggests that *E. repens* rhizome functions not only as a source of bioactive phenolics but also as a matrix rich in hydrophilic compounds that may influence both extract properties and therapeutic action. The mucilaginous matrix may serve to protect volatile and phenolic constituents during preparation and storage and may also facilitate their absorption or interaction with urinary and gastrointestinal tissues [13]. The structures of some carbohydrates found in *E. repens* are shown in Figure 2.

### 5.3. Small Organic Acids and Polar Metabolites

**Figure 3:**
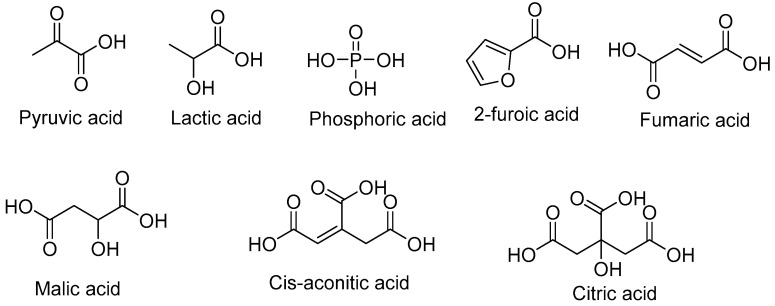
Structures of small organic acid and polar metabolites found in *E. repens*.

Different compounds including pyruvic acid, lactic acid, phosphoric acid, 2-furoic acid, fumaric acid, malic acid, cis-aconitic acid, and citric acid have been reported in *E. repens* (Figure 3) [13,23]. The presence of these organic acids in aqueous extracts contributes to the authentic phytochemical composition of *E. repens* tea and likely influences both the biochemical and organoleptic properties of the preparation. These polar metabolites can be changed to produce acidifying effects sometimes attributed to *E. repens* preparations and may additionally impact the bioavailability and tissue distribution of other active compounds. Many organic acids show inherent biological activity, such as antioxidant properties and potential antibacterial medicinal properties, showing that their use in herbal tea formulations can substantially improve overall pharmacological effectiveness.

### 5.4. Amino Acids and Tryptophan-Related Metabolites

**Figure 4:**
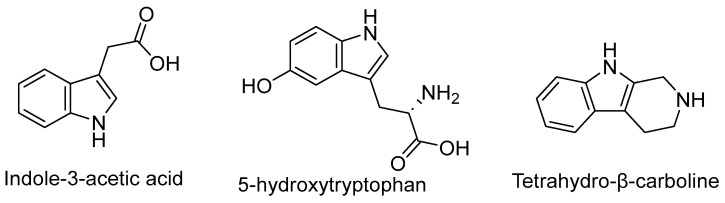
Structures of indole-3-acetic acid, 5-hydroxytryptophan, and tetrahydro-β-carboline.

The rhizome of *E. repens* has a variety of free amino acids, such as GABA (gamma-aminobutyric acid), proline, valine, asparagine, histidine, arginine, and tryptophan. The addition of free amino acids in *E. repens* tea is significant, as these components may enhance the plant’s traditional application as a nutritional tonic. In addition to basic amino acids, the rhizome contains several indole-type metabolites associated with tryptophan metabolism, such as indole-3-acetic acid, 5-hydroxytryptophan, and tetrahydro-β-carboline derivatives (Figure 4). The identification of free tryptophan in *E. repens* rhizome is essential, marking the first report in herbal teas and rhizome extracts, thus extending the chemical profile of the plant beyond traditional urinary treatments [13,32,33]. These findings show a potential physiological significance linked with amino acid metabolism, which is possibly linked to the plant neurochemical pathways involving tryptophan-derived neuromodulators. The current data necessitate additional exploration, since they show the increasing complexity of *E. repens*’ phytochemistry and show that the plant may have biological effects extending beyond its conventional urinary tract properties.

### 5.5. Phenolic Acids and Hydroxycinnamic Acid Derivatives

**Figure 5:**
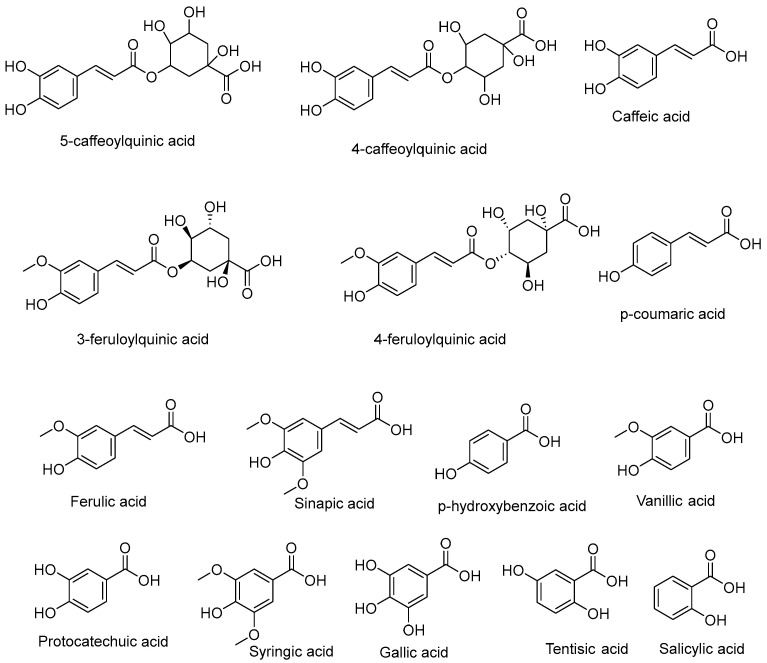
Structures of phenolic acids and hydroxycinnamic acid derivatives in *E. repens*.

Phenolic acids are among the most biologically significant secondary metabolites found in the rhizome, mainly in aqueous formulations. The phytochemical composition is primarily described by hydroxycinnamic acids and quinic esters, which are abundant in caffeoylquinic and feruloylquinic derivatives. The main phenolic acid ingredients comprise 5-caffeoylquinic acid, 4-caffeoylquinic acid, 3-feruloylquinic acid, 4-feruloylquinic acid, caffeic acid, p-coumaric acid, ferulic acid, and sinapic acid. The rhizome also has many phenolic acids, such as p-hydroxybenzoic acid, vanillic acid, protocatechuic acid, syringic acid, gallic acid, gentisic acid, and salicylic acid (Figure 5). These compounds are identified for their antioxidant effects, anti-inflammatory actions, and enzyme inhibition, with some also exhibiting antibacterial and antiadhesive qualities relevant to urinary tract health [13,28,34,35,36,37,38]. The presence of caffeoylquinic and feruloylquinic derivates in such plant rhizomes demonstrates that hydroxycinnamates are important phytochemical markers which show these biological activities. The structural properties also show antioxidant activity and stability in aqueous extract, which further highlights their importance as an essential control indicator for standardization and possible biological activities.

### 5.6. Flavonoids and Flavonoid Glycosides

**Figure 6:**
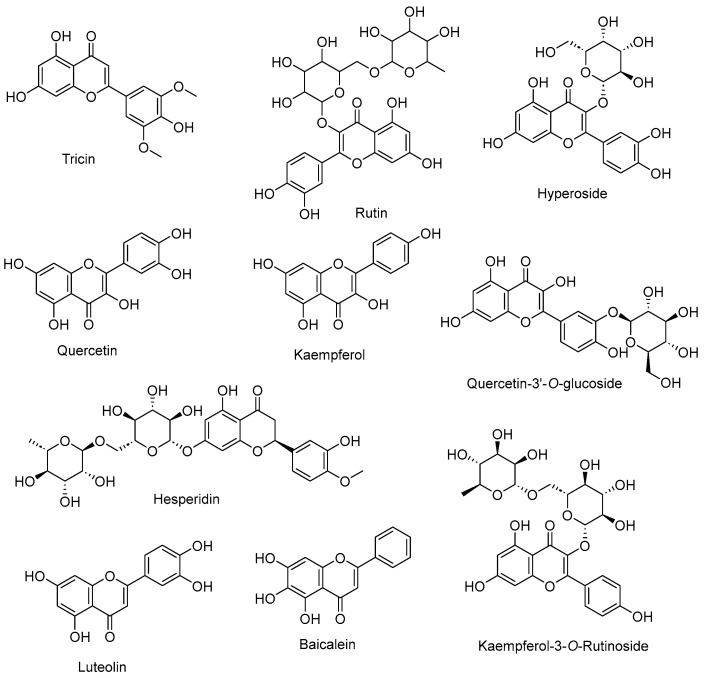
Structures of flavonoids and flavonoid glycosides in *E. repens*.

Flavonoids have been identified in many *E. repens* extracts, with confirmed bioactive compounds tricin, rutin, hyperoside, quercetin, kaempferol, hesperidin, quercetin-3-O-glucoside, kaempferol-3-O-rutinoside, luteolin, and baicalein (Figure 6) [15,24,39,40]. Although quantitatively secondary to phenolic acids in aqueous extracts, flavonoids may synergistically enhance the antioxidant, anti-inflammatory, and enzyme inhibitory activities of the plant. Tricin, a flavone with reported allelopathic properties, needs particular attention due to its potential direct pharmacological importance beyond its function in plant chemistry [41]. The glycosylated flavonoid forms (quercetin glycosides, kaempferol glycosides) are the main compounds present in *E. repens*, and these glycosides may undergo partial hydrolysis during extraction or by gastrointestinal enzymes, resulting in the release of aglycone forms with improved bioavailability. The remaining flavonoids are well-known compounds in plants and are very famous for many biological activities reported in the literature [41,42,43,44,45,46].

### 5.7. Volatile Constituents and Essential Oil Components

**Figure 7:**
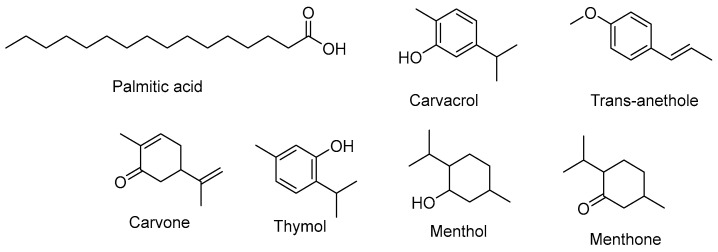
Volatile constituents and essential oil components in *E. repens*.

The essential oil output from *E. repens* rhizome is comparatively low; however, the volatile compounds may still have a substantial role in biological effects, especially in anti-inflammatory and antibacterial activities. The identified volatile compounds contain palmitic acid, carvacrol, trans-anethole, carvone, thymol, menthol, and menthone (Figure 7) [13,23,47]. Instead of their low absolute concentration, the volatile compounds may show disproportionate biological effects due to their potency as antimicrobial and anti-inflammatory agents. In particular, phenolic monoterpenes such as thymol and carvacrol are reported for their strong antimicrobial efficacy [48]. Other compounds such as menthol facilitate anti-inflammatory responses by controlling cytokine production and reducing oxidative stress; they also possess anticancer, antimicrobial, and analgesic properties [49]. Trans-anethole and carvone have also been associated with many biological activities, further improving the therapeutic potential of the volatile fraction [50,51].

### 5.8. Benzoxazinoids and Allelochemicals

**Figure 8:**
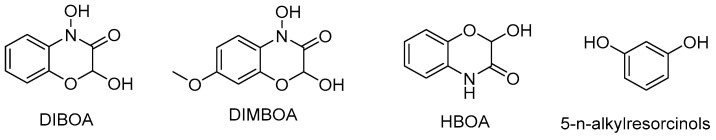
Structures of benzoxazinoids and allelochemicals reported in *E. repens*.

*E. repens* has gained interest as both a medicinal plant and an allelopathic species, as it produces compounds that suppress the growth of adjacent flora. Chemically mediated plant–plant interactions, including allelopathy and allelobiosis, are driven by specialized metabolites (allelochemicals or signaling compounds) released into the environment, influencing the establishment and performance of surrounding vegetation [52]. Benzoxazinoid compounds reported in the rhizome include 2,4-dihydroxy-2H-1,4-benzoxazin-3-one (DIBOA), 2,4-dihydroxy-7-methoxy-2H-1,4-benzoxazin-3-one (DIMBOA), and 2-hydroxy-2H-1,4-benzoxazin-3-one (HBOA) (Figure 8). Additionally, the rhizome contains 5-n-alkylresorcinols, hexadecyl esters of p-hydroxycinnamic acid, and related compounds that may have ecological functions but also add to the plant’s pharmacological complexity. Some of these allelochemicals may participate in plant defense mechanisms or in interfering with microbial colonization, suggesting possible mechanisms for the plant’s reported antimicrobial effects [23,53,54].

A critical distinction must be made regarding certain compounds historically attributed to *E. repens*. Agropyrenol, agropyrenal, and agropyrenone were reported from a fungal pathogen associated with *E. repens*, not from the plant itself, and should therefore not be interpreted as endogenous medicinal constituents of *E. repens*. This important clarification prevents misattribution of bioactivity and ensures that pharmaceutical development focuses on genuinely plant-derived compounds [20,55]. Table 2 shows the above presented major phytochemicals groups, representative compounds and extraction methods of *E. repens* used for it.

## 6. Pharmacological Activities and Therapeutic Mechanisms

### 6.1. Diuretic, Urinary Tract Support, and Antiurolithiatic (Anti-Renal Calculus) Activities

The diuretic and urinary-supportive properties of *E. repens* show its main traditional use, confirmed by further supplementary mechanisms. The rhizome acts as a soothing diuretic, improving urine production while protecting urinary tissues. This effect may include the osmotic properties of glycans and organic acids, mucilage-mediated epithelium relief, anti-inflammatory processes, and antiadhesive activities against urinary pathogens, thus addressing both symptoms and underlying dysfunction [22,23].

*E. repens* shows antiadhesive properties against uropathogenic *Escherichia coli* (UPEC), hence supporting urinary tract health. An acetone extract primarily inhibited bacterial adherence to human T24 bladder cells with no cytotoxicity; the active compound was found to be (E)-hexadecyl-3-(4-hydroxyphenyl)-acrylate that reduces both adhesion and invasion [56]. This suggests a preventive mechanism against infection establishment.

A pediatric randomized controlled study (*n* = 50) on urinary stone disease revealed that *E. repens* extract dramatically decreased the quantity (88%) and size (92%) of renal calculi, while also improving renal colic, hematuria, and sepsis, without severe adverse effects [57]. These effects are mainly due to the increased urine flow, anti-inflammatory activity, antimicrobial support, and spasmolytic action.

**Figure 9:**
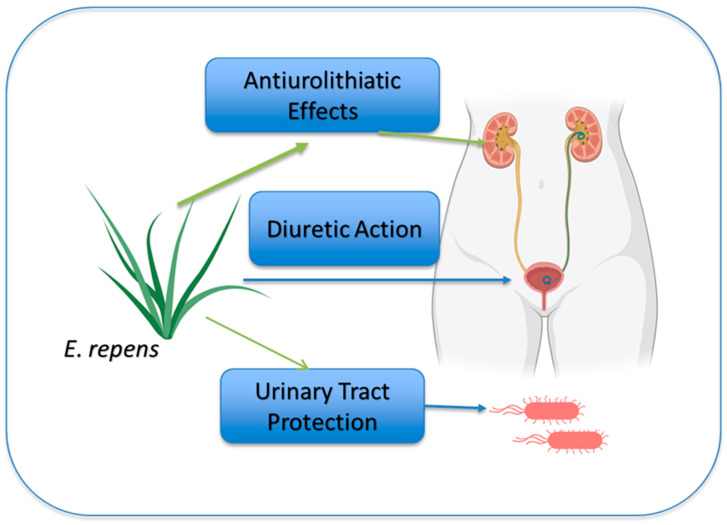
Overview of the diuretic, urinary tract-protective, and antiurolithiatic activities of *E. repens*.

Overall, *E. repens* shows multifaceted biological activities, combining diuretic, antiurolithiatic, and urinary-protective effects (Figure 9), although further careful clinical validation is needed.

### 6.2. Hypoglycemic and Antidiabetic Activity

*E. repens* exhibited both direct hypoglycemic effects relevant to the management of diabetes and modifications in carbohydrate digestion. In an in vivo study, the aqueous extract reduced glucose levels in both normal and streptozotocin-induced diabetic rats, normalized with repeated administration and its effect seemed independent of insulin secretion, suggesting a non-insulin-dependent glucose regulatory mechanism. These data suggest potential effects on hepatic glucose production, peripheral glucose absorption, or metabolic remodeling that are independent of beta-cell function [14]. In a similar way, in vitro investigation of hydroalcoholic extract displayed the inhibition of α-amylase and α-glucosidase, crucial enzymes in controlling diabetes. These inhibitory effects indicate that plants may reduce the rate and amount of carbohydrate absorption, thereby reducing postprandial glucose variability, similar to the mechanism of the standard drug acarbose [17]. Other enzymes such as PTP1B and DPP4 are also related to diabetes and can be studied in a similar way [58,59].

*E. repens* can reduce systemic glucose and subsequently reduce the digestive enzymes that regulate glucose. The plant contains phenolic acids, flavonoids, and hydroxycinnamate derivatives that may cause these effects. This dual mechanism suggests that *E. repens* may benefit fasting and subsequent glucose dysregulation. Further research should identify the active components or biological compounds, quantify the dose–response relationships, and evaluate the effects in relevant animal models and human subjects with different types of diabetes phenotypes.

### 6.3. Anti-Inflammatory Activity

The anti-inflammatory properties of *E. repens* have been reported and are chemically confirmed by the plant’s identified phenolic acids, flavonoids, and volatile compounds. These anti-inflammatory effects may be facilitated through antioxidant-related inflammatory suppression, possible modulation of pro-inflammatory mediators such as cytokines and chemokines, and contribution of hydroxycinnamic derivatives and tricin-like flavones with known anti-inflammatory properties. However, the underlying molecular pathways remain insufficiently studied, with most available confirmation consisting of relatively general anti-inflammatory assays instead of mechanistic investigations targeting specific inflammatory cascades [15,60]. Future research should utilize modern approaches including cytokine profiling, transcriptomic analysis, and investigation of specific inflammatory pathways relevant to conditions where *E. repens* is traditionally used.

### 6.4. Antioxidant Activity

The antioxidant capacity of *E. repens* has been reported by several assay systems, including DPPH radical scavenging and reducing power performed on hydroalcoholic extracts. The antioxidant activity directly correlates with present phenolic and flavonoid content, particularly caffeoylquinic acids, feruloylquinic acids, caffeic acid, and flavonoid constituents. The antioxidant capacity of *E. repens* probably contributes significantly to its broader anti-inflammatory, metabolic, and urinary tract protective effects, as oxidative stress represents a common mechanism underlying multiple pathological conditions [15,17,24]. While antioxidant activity does not indicate a primary therapeutic application of the plant, it likely highlights and supports the plant’s effects on other conditions for which oxidative stress represents a pathogenic factor.

### 6.5. Phytochemical–Pharmacological Correlations

**Figure 10:**
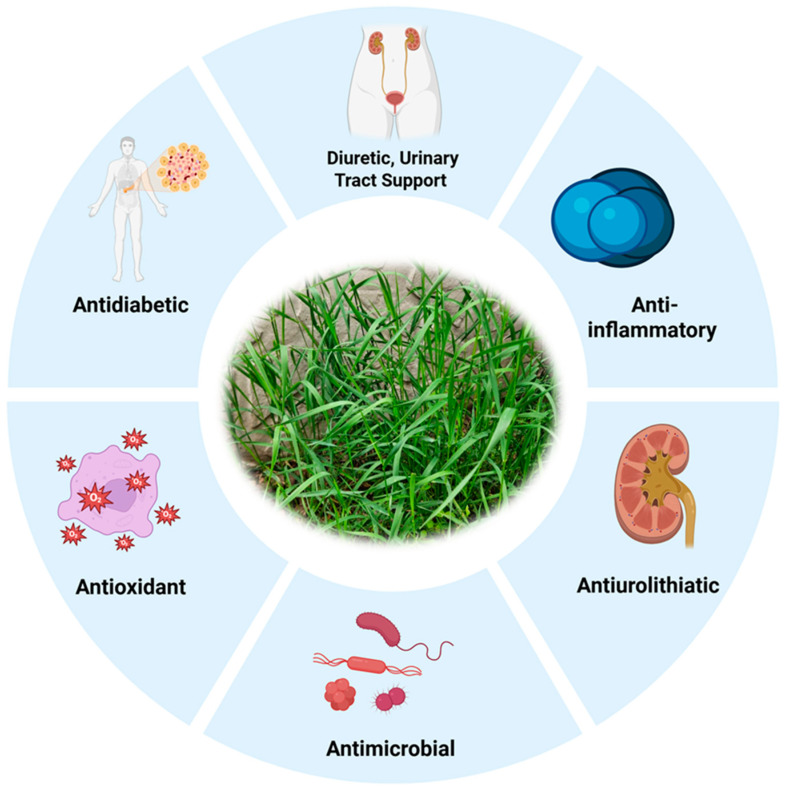
Biological activities of *E. repens*.

Based on the above Section 6.1, Section 6.2, Section 6.3 and Section 6.4, the pharmacological properties of *E. repens* are mainly facilitated by synergistic interactions among multiple phytochemical classes instead of a single major constituent. Previous studies have reported that *E. repens* exhibits diverse biological activities (Figure 10). Phenolic acids and their derivatives influence antioxidants and anti-inflammatory effects through radical scavenging and enzyme modulation. Polyphenols and flavonoids appear responsible for enzyme inhibition relevant to antidiabetic activity. Saccharides, fructans, and mucilaginous compounds support soothing and demulcent effects on urinary mucosa. Volatile constituents and certain allelochemicals contribute antimicrobial and antiadhesive effects. This polyvalent pharmacological profile displays a distinctive feature of complex botanical preparations compared to single-compound pharmaceuticals, and it may underline the clinical effectiveness of traditional *E. repens* preparations. The overall biological activities of *E. repens* are illustrated in Figure 10.

## 7. Pharmaceutical Relevance and Clinical Potential

### 7.1. Significance of Herbal Tea and Aqueous Preparations

Although many phytochemical studies have investigated non-aqueous extracts, herbal tea remains the traditional and most practically relevant medicinal form. Recent compound profiling of the rhizome herbal tea has demonstrated that aqueous preparations contain a significant number of compounds, including phenolic acids, amino acids, and saccharides. This finding verifies traditional preparation methods and links folk practice to modern phytochemistry. The aqueous preparations are clinically advantageous because of their accessibility; also it is cost effective and has the potential for evaluating the biological structure of traditional treatments [13].

### 7.2. Safety, Toxicity and Quality Considerations

*E. repens* has a long history of traditional use without any clear side effects, which establishes a strong foundation for further pharmacological development. However, there is still a gap present in terms of toxicity, which should be acknowledged so there is no clear toxicity profile; also, the chronic toxicity, reproductive and genotoxicity studies are inadequate and complete herb drug interaction studies have not been conducted. The absence of knowledge does not necessarily indicate that the plant is unsafe, rather it indicates that a thorough safety assessment according to established criteria has not been conducted.

There are potential concerns regarding *E. repens* including the presence of allelochemicals [61,62], their clear biological functions, phytochemical variations related to harvest season and geographical origin, different quality of raw materials between different sources, and unclear information about certain minor constituents and their effects. In regard to pharmacological perspective, the main problem for *E. repens* lies in both the validation of efficacy and the development of reliable quality control parameters for chemically diverse rhizome preparations. However, confirmed commercial samples have generally demonstrated acceptable microbiological quality and low water activity, suggesting that effective quality control is achievable [63,64].

### 7.3. Standardization Challenges

Another main challenge with *E. repens* is standardization, starting from the chemical complexity of the plant and environmental sensitivity. Along with this, harvest time influences the phytochemical composition, because different seasons provide different chemical profiles [65,66]. Geographic origins also change the composition, reflecting edaphic and climatic factors [67,68]. Along with this, drying and storage techniques affect the preservation of unstable molecules and oxidation-sensitive substances [69]. Also, the extraction method, which mostly shows the compound’s makeup and bioactivity of the final product, is another concern [70]. These characteristics presented from different studies show that *E. repens* produces products from different producers, or different batches from the same manufacturer can show significant heterogeneity in the composition of the compounds and the expected biological activity.

## 8. Current Evidence and Research Gaps

The clinical evidence for *E. repens* is generally small, with the most noted contribution appearing from the previously stated pediatric perspective-controlled trial on renal calculus reduction [57]. This single investigation shows that significant therapeutic outcomes can be achieved; however, additional detailed randomized controlled trials are necessary to establish *E. repens* as an evidence-based pharmaceutical treatment. The ability to draw significant conclusions about efficacy, ideal dosage, duration of treatment, and suitability for specific patient populations is limited by the absence of multiple comprehensive clinical trials. Other important research gaps include the identification of active compounds associated with reported pharmacological effects, limited mechanistic investigation of the molecular and cellular pathways underlying bioactivity, limited pharmacokinetic data on absorption, distribution, metabolism, and excretion of active ingredients, lack of extracts available for quality research or standardized extracts for clinical application, and inadequate toxicity assessment according to modern safety standards. Research priorities include bioassay-guided isolation of active compounds, focused investigations on anti-urolithiasis mechanisms of stone formation, mechanistic analysis of anti-diabetic pathways, development of standardized markers from phenolic acids and tryptophan, as well as molecular docking and network pharmacology, which describe detailed interactions between compounds and targets [71]. Special importance should be directed towards research studying aqueous formulations, as these represent the standard and pragmatic dosage form.

## 9. Conclusions

In this review, we carefully presented the complex medicinal plant *E. repens*, which has many ethnopharmacological properties. Traditional uses such as in urinary tract disorders are confirmed by increasing pharmacological and preliminary clinical evidence demonstrating diuretic, anti-urolithic, anti-inflammatory, antioxidant, and metabolic regulatory effects. The phytochemicals present in this plant having a synergistic effect mainly include polysaccharides, phenolics, flavonoids, and benzoxazinoids, which suggests that its efficacy arises from integrated, multi-targeted mechanisms rather than the actions of individual compounds. Despite these fruitful results, the current data are limited by insufficient clinical validation, absence of standardized extract, and poor understanding of structure–activity interactions. Addressing these possible research gaps requires an interdisciplinary approach that combines phytochemistry, pharmacology and clinical research. Also, future research should include detailed clinical trials, advanced metabolomic data and mechanistic investigation to confirm the efficacy and reproducibility. With appropriate standardization and scientific confirmation, *E. repens* holds a considerable position for development as a safe and effective therapeutic agent.

## Figures and Tables

**Table 1:** Botanical identity and traditional uses of *E. repens*.

Feature	Description
Accepted Name	*Elymus repens* (L.) Gould
Synonyms	*Elytrigia repens* (L.); *Agropyron repens* (L.) P. Beauv.; *Triticum repens* (L.)
Family	Poaceae
Common Names	Couch grass, quackgrass, wheatgrass
Distribution	Native to temperate Europe and Central Asia; now widespread globally
Medicinal Part	Rhizome
Traditional Preparation	Infusion, decoction, herbal tea (aqueous extraction)
Traditional Uses	Diuretic, demulcent, urinary tract remedy, tonic, antilithic

**Table 2:** Different phytochemical classes with their representative compounds and extract types of *E. repens*.

Class	Representative Compounds	Extract Type
Fructans/Carbohydrates	Triticin, fructosan, inulin, glucose, fructose	Water
Phenolic Acids	Caffeoylquinic acids, feruloylquinic acids, caffeic acid	Water, hydroalcoholic
Flavonoids	Tricin, rutin, quercetin, kaempferol	Hydroalcoholic, water
Amino Acids	Tryptophan, GABA, proline, valine	Water
Volatile Compounds	Carvacrol, thymol, menthol, carvone	Essential oil
Benzoxazinoids	DIBOA, DIMBOA, alkylresorcinols	Organic solvents

## Data Availability

All data regarding this review article are available in this manuscript and no further data have been generated.

## References

[1] Davis C.C., Choisy P. (2024). Medicinal Plants Meet Modern Biodiversity Science. Curr. Biol..

[2] Martínez-González R.E., Huerta-Martínez F.M., Neri-Luna C., Barrientos-Ramírez L., Muñoz-Urias A. (2025). Ethnobotany in a Modern City: The Persistence in the Use of Medicinal Plants in Guadalajara, Mexico. Plants.

[3] Shah A.B., Kim Y.J., Lee K.S., Han S.H., Byun Y., Lee K.Y. (2026). Natural Products Modulating Interleukin-Mediated Pathways for Anti-Allergic and Immunomodulatory Effects. Nat. Prod. Rep..

[4] Najmi A., Javed S.A., Al Bratty M., Alhazmi H.A. (2022). Modern Approaches in the Discovery and Development of Plant-Based Natural Products and Their Analogues as Potential Therapeutic Agents. Molecules.

[5] Wang H., Chen Y., Wang L., Liu Q., Yang S., Wang C. (2023). Advancing Herbal Medicine: Enhancing Product Quality and Safety through Robust Quality Control Practices. Front. Pharmacol..

[6] Nguyen A.N.T., Vu T.T.T., Do H.T.T., Nguyen T.H., Le H.V., Pham H.K.T., Truong P.C.H., Pham D.P., Tran M.H. (2024). Identification of Phenolic Compounds from Vietnamese Artichoke(*Cynara scolymus* L.) Leaf and Their Antioxidant Activities. Nat. Prod. Sci..

[7] Jang J.S., Han J.S., Cho Y.B., An B.K., Hwang B.Y., Lee M.-S. (2024). Anti-Inflammatory Activity of Norisoprenoids from the Aerial Parts of *Celosia cristata* L.. Nat. Prod. Sci..

[8] Kim H.R., Lee G.S., Park I., Kim C.S. (2024). Indole Derivatives and a Diketopiperazine from *Chromobacterium violaceum*. Nat. Prod. Sci..

[9] Salm S., Rutz J., van den Akker M., Blaheta R.A., Bachmeier B.E. (2023). Current State of Research on the Clinical Benefits of Herbal Medicines for Non-Life-Threatening Ailments. Front. Pharmacol..

[10] Ali S., Khalil A.A.K., Akhtar M.S., Amin A., Zaman W. (2026). Comprehensive Insights into Natural Bioactive Compounds: From Chemical Diversity and Mechanisms to Biotechnological Innovations and Applications. ChemistryOpen.

[11] Chaachouay N., Zidane L. (2024). Plant-Derived Natural Products: A Source for Drug Discovery and Development. Drugs Drug Candidates.

[12] Ringselle B., Cauwer B.D., Salonen J., Soukup J. (2020). A Review of Non-Chemical Management of Couch Grass (*Elymus repens*). Agronomy.

[13] Bortolami M., Di Matteo P., Rocco D., Feroci M., Petrucci R. (2022). Metabolic Profile of *Agropyron repens* (L.) *P. beauv*. Rhizome Herbal Tea by HPLC-PDA-ESI-MS/MS Analysis. Molecules.

[14] Eddouks M., Maghrani M., Michel J.B. (2005). Hypoglycaemic Effect of Triticum Repens *P. beauv*. in Normal and Diabetic Rats. J. Ethnopharmacol..

[15] Petrova A.P., Krasnov E.A., Saprykina E.V., Subbotina Y.A., Ermilova E.V. (2009). Chemical Composition of Couch Grass and Studies of Its Antioxidant Activity in Allergic Contact Dermatitis. Pharm. Chem. J..

[16] Hautmann C., Scheithe K. (2000). Fluid extract of *Agropyron repens* for the treatment of urinary tract infections or irritable bladder. Results of multicentric post-marketing surveillance. Z. Phytother..

[17] Elena N., Gabriela P., Veronica M., Oana U., Gabriel L.R. (2019). Antioxidant and Antidiabetic Properties of Polyphenolic-Rich Extracts of *Apium graveolens* and *Agropyrum repens*. Rev. Roum. Chim..

[18] *Elytrigia repens*|Federal Noxious Weed Disseminules of the U.S. https://idtools.org/fnwd/index.cfm?packageID=1097&entityID=2583.

[19] NAL Agricultural Thesaurus: NALT: *Elymus repens* Subsp. Repens. https://lod.nal.usda.gov/nalt/en/page/135155.

[20] Andolfi A., Cimmino A., Vurro M., Berestetskiy A., Troise C., Zonno M.C., Motta A., Evidente A. (2012). Agropyrenol and Agropyrenal, Phytotoxins from *Ascochyta agropyrina* Var. Nana, a Fungal Pathogen of *Elitrigia repens*. Phytochemistry.

[21] Korhammer S.A., Haslinger E. (1994). Isolation of a Biologically Active Substance from Rhizomes of Quackgrass [*Elymus repens* (L.) Gould). J. Agric. Food Chem..

[22] Jamshaid M., Rashid U., Butt Z.A., Munazir M., Qureshi R. (2022). Phytochemical Analysis of Methanolic Extracts of *Elymus repens*, *Typha angustifolia* and *Caralluma edulis*. Open Access Res. J. Biol. Pharm..

[23] Al-Snafi A.E. (2015). Chemical Constituents and Pharmacological Importance of *Agropyron repens*—A Review. Res. J. Pharmacol. Toxicol..

[24] Deveci E., Tel Çayan G., Karakurt S., Duru M. (2020). Antioxidant, Cytotoxic, and Enzyme Inhibitory Activities of *Agropyron repens* and Crataegus Monogyna Species. Eur. J. Biol..

[25] Ringselle B., Brandsæter L.O., Mangerud K., Bergkvist G. (2023). Vertical Rhizome Disking to Reduce *Elymus repens* (Quackgrass) Abundance in Grass-Clover Leys. Crop Prot..

[26] *Elymus repens* (AGRRE)[Overview]|EPPO Global Database. https://gd.eppo.int/taxon/AGRRE.

[27] Andreasen C., Vlassi E., Salehan N. (2024). Laser Weeding: Opportunities and Challenges for Couch Grass (*Elymus repens* (L.) Gould) Control. Sci. Rep..

[28] Beydokthi S.S., Sendker J., Brandt S., Hensel A. (2017). Traditionally Used Medicinal Plants against Uncomplicated Urinary Tract Infections: Hexadecyl Coumaric Acid Ester from the Rhizomes of *Agropyron repens* (L.) *P. beauv.* with Antiadhesive Activity against Uropathogenic *E. coli*. Fitoterapia.

[29] Nirumand M.C., Hajialyani M., Rahimi R., Farzaei M.H., Zingue S., Nabavi S.M., Bishayee A. (2018). Dietary Plants for the Prevention and Management of Kidney Stones: Preclinical and Clinical Evidence and Molecular Mechanisms. Int. J. Mol. Sci..

[30] Kasote D.M., Jagtap S.D., Thapa D., Khyade M.S., Russell W.R. (2017). Herbal Remedies for Urinary Stones Used in India and China: A Review. J. Ethnopharmacol..

[31] Mueller S.O., Schmitt M., Dekant W., Stopper H., Schlatter J., Schreier P., Lutz W.K. (1999). Occurrence of Emodin, Chrysophanol and Physcion in Vegetables, Herbs and Liquors. Genotoxicity and Anti-Genotoxicity of the Anthraquinones and of the Whole Plants. Food Chem. Toxicol..

[32] Hagin R.D. (1989). Isolation and Identification of 5-Hydroxyindole-3-Acetic Acid and 5-Hydroxytryptophan, Major Allelopathic Aglycons in Quackgrass (*Agropyron repens* L. Beauv.). J. Agric. Food Chem..

[33] Hagin R.D., Bobnick S.J. (1991). Isolation and Identification of a Slug-Specific Molluscicide from Quack Grass (*Agropyron repens*, L. Beauv.). J. Agric. Food Chem..

[34] Koetter U., Kaloga M., Schilcher H. (2007). Isolierung and Strukturaufklärung von p-Hydroxyzimtsäurealkylester-Verbindungen aus dem Rhizom von *Agropyron repens*; 1. Mitteilung. Planta Med..

[35] Koetter U., Kaloga M., Schilcher H. (1994). Isolation and Structure Elucidation of P-Hydroxycinnamic Acid Esters from the Rhizom of *Agropyron repens*, Part II. Planta Med..

[36] Espíndola K.M.M., Ferreira R.G., Narvaez L.E.M., Silva Rosario A.C.R., da Silva A.H.M., Silva A.G.B., Vieira A.P.O., Monteiro M.C. (2019). Chemical and Pharmacological Aspects of Caffeic Acid and Its Activity in Hepatocarcinoma. Front. Oncol..

[37] Sova M., Saso L. (2020). Natural Sources, Pharmacokinetics, Biological Activities and Health Benefits of Hydroxycinnamic Acids and Their Metabolites. Nutrients.

[38] Wang L., Pan X., Jiang L., Chu Y., Gao S., Jiang X., Zhang Y., Chen Y., Luo S., Peng C. (2022). The Biological Activity Mechanism of Chlorogenic Acid and Its Applications in Food Industry: A Review. Front. Nutr..

[39] Weston L.A., Burke B.A., Putnam A.R. (1987). Isolation, Characterization and Activity of Phytotoxic Compounds from Quackgrass [*Agropyron repens* (L.)Beauv]. J. Chem. Ecol..

[40] Dhanalakshmi K., Bhavan P.S., Rajkumar G., Nathiya V., Srinivasan V., Satgurunathan T. (2016). Phytochemical Characterization of Couch Grass (*Cynodon dactylon*) and Its Growth Promoting Potential on the Freshwater Prawn Macrobrachium Rosenbergii Post-Larvae. Biotechnol. J. Int..

[41] Li X.-X., Chen S.-G., Yue G.G.-L., Kwok H.-F., Lee J.K.-M., Zheng T., Shaw P.-C., Simmonds M.S.J., Lau C.B.-S. (2021). Natural Flavone Tricin Exerted Anti-Inflammatory Activity in Macrophage via NF-κB Pathway and Ameliorated Acute Colitis in Mice. Phytomedicine.

[42] Shah A.B., Yoon S., Kim J.H., Zhumanova K., Ban Y.J., Lee K.W., Park K.H. (2020). Effectiveness of Cyclohexyl Functionality in Ugonins from *Helminthostachys zeylanica* to PTP1B and α-Glucosidase Inhibitions. Int. J. Biol. Macromol..

[43] Zhumanova K., Lee G., Baiseitova A., Shah A.B., Kim J.H., Kim J.Y., Lee K.W., Park K.H. (2021). Inhibitory Mechanism of O-Methylated Quercetins, Highly Potent β-Secretase Inhibitors Isolated from *Caragana balchaschensis* (Kom.) Pojark. J. Ethnopharmacol..

[44] Periferakis A., Periferakis K., Badarau I.A., Petran E.M., Popa D.C., Caruntu A., Costache R.S., Scheau C., Caruntu C., Costache D.O. (2022). Kaempferol: Antimicrobial Properties, Sources, Clinical, and Traditional Applications. Int. J. Mol. Sci..

[45] Salimi A., Asgari B., Khezri S., Pourgholi M., Haddadi S. (2025). Hesperidin as a Bioactive Compound in Citrus Fruits Reduces N-Ethyl-N-Nitrosourea-Induced Mortality and Toxicity in Mice: As a Model for Chronic Lymphocytic Leukemia. Naunyn Schmiedeberg’s Arch. Pharmacol..

[46] Lin T.-S., Cai X.-X., Wang Y.-B., Xu J.-T., Xiao J.-H., Huang H.-Y., Li S.-F., Liu K.-M., Chen J.-H., Li L.-P. (2025). Identifying Baicalein as a Key Bioactive Compound in XueBiJing Targeting KEAP1: Implications for Antioxidant Effects. Antioxidants.

[47] Boesel R., Schilcher H. (1989). Composition of the Essential Oil of *Agropyrum repens* Rhizome1. Planta Med..

[48] Radocchia G., Giammarino A., Barberini S., Verdolini L., De Angelis M., Simonetti G., Pantanella F., Schippa S., Angiolella L. (2025). Carvacrol and Thymol, a Synergistic Antimicrobial Activity Against Bacterial and Candida Species. Microbiologyopen.

[49] Zhang J., Hu Y., Wang Z. (2025). Menthol and Its Derivatives: Exploring the Medical Application Potential. Eng. Life Sci..

[50] Bouyahya A., Mechchate H., Benali T., Ghchime R., Charfi S., Balahbib A., Burkov P., Shariati M.A., Lorenzo J.M., Omari N.E. (2021). Health Benefits and Pharmacological Properties of Carvone. Biomolecules.

[51] Raposo A., Raheem D., Zandonadi R.P., Suri N., Olukosi A., de Lima B.R., Carrascosa C., Sharifi-Rad J., Ryu H.B., Han H. (2024). Anethole in Cancer Therapy: Mechanisms, Synergistic Potential, and Clinical Challenges. Biomed. Pharmacother..

[52] Kong C.-H., Li Z., Li F.-L., Xia X.-X., Wang P. (2024). Chemically Mediated Plant–Plant Interactions: Allelopathy and Allelobiosis. Plants.

[53] Macías F.A., Oliveros-Bastidas A., Marín D., Chinchilla N., Castellano D., Molinillo J.M.G. (2014). Evidence for an Allelopathic Interaction Between Rye and Wild Oats. J. Agric. Food Chem..

[54] Kubus G., Tłuścik F. (1983). Alkyl Resorcinols in Grains from Plants from the Family Gramineae. Acta Soc. Bot. Pol..

[55] Cimmino A., Zonno M.C., Andolfi A., Troise C., Motta A., Vurro M., Evidente A. (2013). Agropyrenol, a Phytotoxic Fungal Metabolite, and Its Derivatives: A Structure–Activity Relationship Study. J. Agric. Food Chem..

[56] Sarshar S., Hensel A. (2015). Antiadhesive Effect of *Agropyron repens* L. Rhizome Extract against Uropathogenic *E. coli* and Pinpointing (E)-Hexadecyl 3-(4-Hydroxyphenyl) Acrylate as Active Ingredient. Planta Med..

[57] Moohy Alosy B.D., Thakir E.M., Khalaf S.A. (2019). Role of *Agropyron repens* Extract in Treatment Renal Calculus in Pediatric Group. Indian J. Forensic Med. Toxicol..

[58] Röhrborn D., Wronkowitz N., Eckel J. (2015). DPP4 in Diabetes. Front. Immunol..

[59] Shah A.B., Baiseitova A., Lee G., Kim J.H., Park K.H. (2024). Analogues of Dihydroflavonol and Flavone as Protein Tyrosine Phosphatase 1B Inhibitors from the Leaves of *Artocarpus elasticus*. ACS Omega.

[60] Mascolo N., Autore G., Capasso F., Menghini A., Fasulo M.P. (1987). Biological Screening of Italian Medicinal Plants for Anti-Inflammatory Activity. Phytother. Res..

[61] Quan X., Qiao Y., Chen M., Duan Z., Shi H. (2021). Comprehensive Evaluation of the Allelopathic Potential of Elymus Nutans. Ecol. Evol..

[62] Glinwood R., Pettersson J., Ahmed E., Ninkovic V., Birkett M., Pickett J. (2003). Change in Acceptability of Barley Plants to Aphids After Exposure to Allelochemicals from Couch-Grass (*Elytrigia repens*). J. Chem. Ecol..

[63] Myemba D.T., Bwire G.M., Sangeda R.Z. (2022). Microbiological Quality of Selected Local and Imported Non-Sterile Pharmaceutical Products in Dar Es Salaam, Tanzania. Infect. Drug Resist..

[64] Ichim M.C., Booker A. (2021). Chemical Authentication of Botanical Ingredients: A Review of Commercial Herbal Products. Front. Pharmacol..

[65] Ghimire B.K., Seo J.-W., Kim S.-H., Ghimire B., Lee J.-G., Yu C.-Y., Chung I.-M. (2021). Influence of Harvesting Time on Phenolic and Mineral Profiles and Their Association with the Antioxidant and Cytotoxic Effects of *Atractylodes japonica* Koidz. Agronomy.

[66] Kołtun-Jasion M., Kicel A., Hińczewska M., Dudek M.K., Olszewska M., Kiss A.K. (2026). From Spring to Autumn: How Harvest Season and Species Shape the Phytochemical and Biological Properties of Forsythia Leaf Extracts. Ind. Crops Prod..

[67] van Staden N., van der Merwe H., Siebert S. (2026). Edaphic and Climatic Drivers of Herbaceous Plant Diversity in Geologically Distinct Mountain Floras of Griqualand West, South Africa. S. Afr. J. Bot..

[68] Kosanic A., Anderson K., Harrison S., Turkington T., Bennie J. (2018). Changes in the Geographical Distribution of Plant Species and Climatic Variables on the West Cornwall Peninsula (South West UK). PLoS ONE.

[69] Babarabie M., Mohammadi M., Ghorbanzadeh A., Afsharipour S., Salari F. (2025). Effect of Drying Conditions on the Preservation of Selected Bioactive Compounds in Moringa Oleifera Aqueous Extract: Acetic Acid, Butyric Acid, γ-Aminobutyric Acid, Salicin, and Glycine. BMC Plant Biol..

[70] Sun S., Yu Y., Jo Y., Han J.H., Xue Y., Cho M., Bae S.-J., Ryu D., Park W., Ha K.-T. (2025). Impact of Extraction Techniques on Phytochemical Composition and Bioactivity of Natural Product Mixtures. Front. Pharmacol..

[71] Shah A.B., Lee K.Y. (2025). Integrative Metabolomics and System Pharmacology Reveal the Antioxidant Blueprint of *Psoralea corylifolia*. Sci. Rep..

